# Supplementary material for: Molecular phylogenetics and evolutionary history of the endemic land snail genus Everettia in northern Borneo
Source: PeerJ. 2020 Jul 9;8:e9416. doi: 10.7717/peerj.9416 (PMC7354840; doi:10.7717/peerj.9416)
Supplement: Supplemental Information 2 — Each of the six partitions, namely, codons of COI, namely, 1st, 2nd and 3rd codon positions of COI, 16S rDNA, ITS-1 and 28S rDNA, was tested for molecular evolution via ModelFinder (Kalyaanamoorthy et al., 2017) and partition models (Chernomor, Von Haeseler & Minh, 2016) based on the both AIC and BIC that built into IQ-Tree v.1.6.7 (Nguyen et al., 2015; Trifinopoulos et al., 2016). We limited the candidate models to the six models that are available in MrBayes analysis, namely, JC, F81, K80, HKY, SYM and GTR. Besides, parameters used in BEAST analysis for divergence time estimation were included. [file peerj-08-9416-s002.docx]

28S

Model selected:

Model = HKY+I

partition = 010010

-lnL = 1481.2745

K = 99

freqA = 0.2535

freqC = 0.2502

freqG = 0.3087

freqT = 0.1876

kappa = 2.6142 (ti/tv = 1.3297)

p-inv = 0.9290

--

PAUP* Commands Block:

If you want to load the selected model and associated estimates in PAUP*,

attach the next block of commands after the data in your PAUP file:

[!

Likelihood settings from best-fit model (HKY+I) selected by AIC

with jModeltest 2.1.10 v20160303 on Thu Jul 19 18:52:01 PDT 2018]

BEGIN PAUP;

Lset base=(0.2535 0.2502 0.3087 ) nst=2 tratio=1.3297 rates=equal pinvar=0.9290;

END;

--

16S

Model selected:

Model = GTR+I+G

partition = 012345

-lnL = 4998.0343

K = 140

freqA = 0.4141

freqC = 0.1074

freqG = 0.1197

freqT = 0.3587

R(a) [AC] = 0.6729

R(b) [AG] = 6.6843

R(c) [AT] = 2.4115

R(d) [CG] = 0.5637

R(e) [CT] = 3.5806

R(f) [GT] = 1.0000

p-inv = 0.1900

gamma shape = 0.3390

--

PAUP* Commands Block:

If you want to load the selected model and associated estimates in PAUP*,

attach the next block of commands after the data in your PAUP file:

[!

Likelihood settings from best-fit model (GTR+I+G) selected by AIC

with jModeltest 2.1.10 v20160303 on Thu Jul 19 18:56:49 PDT 2018]

BEGIN PAUP;

Lset base=(0.4141 0.1074 0.1197 ) nst=6 rmat=(0.6729 6.6843 2.4115 0.5637 3.5806) rates=gamma shape=0.3390 ncat=4 pinvar=0.1900;

END;

COI123

PAUP* Commands Block:

If you want to load the selected model and associated estimates in PAUP*,

attach the next block of commands after the data in your PAUP file:

[!

Likelihood settings from best-fit model (HKY+I+G) selected by BIC

with jModeltest 2.1.10 v20160303 on Wed Jun 27 17:40:02 PDT 2018]

BEGIN PAUP;

Lset base=(0.3016 0.0842 0.1386 ) nst=2 tratio=4.2721 rates=gamma shape=0.6090 ncat=4 pinvar=0.5130;

END;

COI1--

Model selected:

Model = TrN+I+G

partition = 010020

-lnL = 903.1276

K = 145

freqA = 0.2759

freqC = 0.1287

freqG = 0.2989

freqT = 0.2964

R(a) [AC] = 1.0000

R(b) [AG] = 2.1743

R(c) [AT] = 1.0000

R(d) [CG] = 1.0000

R(e) [CT] = 56.5968

R(f) [GT] = 1.0000

p-inv = 0.4590

gamma shape = 0.6000

--

PAUP* Commands Block:

If you want to load the selected model and associated estimates in PAUP*,

attach the next block of commands after the data in your PAUP file:

[!

Likelihood settings from best-fit model (TrN+I+G) selected by AIC

with jModeltest 2.1.10 v20160303 on Thu Jul 19 18:54:37 PDT 2018]

BEGIN PAUP;

Lset base=(0.2759 0.1287 0.2989 ) nst=6 rmat=(1.0000 2.1743 1.0000 1.0000 56.5968) rates=gamma shape=0.6000 ncat=4 pinvar=0.4590;

END;

COI2

Model selected:

Model = TVM+I

partition = 012314

-lnL = 339.9084

K = 146

freqA = 0.1200

freqC = 0.2429

freqG = 0.1844

freqT = 0.4527

R(a) [AC] = 2035.9781

R(b) [AG] = 562.3539

R(c) [AT] = 780.4383

R(d) [CG] = 1227.7834

R(e) [CT] = 562.3539

R(f) [GT] = 1.0000

p-inv = 0.7750

--

PAUP* Commands Block:

If you want to load the selected model and associated estimates in PAUP*,

attach the next block of commands after the data in your PAUP file:

[!

Likelihood settings from best-fit model (TVM+I) selected by AIC

with jModeltest 2.1.10 v20160303 on Thu Jul 19 18:56:38 PDT 2018]

BEGIN PAUP;

Lset base=(0.1200 0.2429 0.1844 ) nst=6 rmat=(2035.9781 562.3539 780.4383 1227.7834 562.3539) rates=equal pinvar=0.7750;

END;

--

COI12

Model selected:

Model = TIM1+I+G

partition = 012230

-lnL = 1335.4470

K = 146

freqA = 0.2037

freqC = 0.1965

freqG = 0.2403

freqT = 0.3594

R(a) [AC] = 1.0000

R(b) [AG] = 3.8131

R(c) [AT] = 2.2129

R(d) [CG] = 2.2129

R(e) [CT] = 27.8670

R(f) [GT] = 1.0000

p-inv = 0.6370

gamma shape = 0.4090

--

PAUP* Commands Block:

If you want to load the selected model and associated estimates in PAUP*,

attach the next block of commands after the data in your PAUP file:

[!

Likelihood settings from best-fit model (TIM1+I+G) selected by AIC

with jModeltest 2.1.10 v20160303 on Thu Jul 19 18:58:12 PDT 2018]

BEGIN PAUP;

Lset base=(0.2037 0.1965 0.2403 ) nst=6 rmat=(1.0000 3.8131 2.2129 2.2129 27.8670) rates=gamma shape=0.4090 ncat=4 pinvar=0.6370;

END;

--

COI3

Model selected:

Model = TIM1+G

partition = 012230

-lnL = 5129.3146

K = 145

freqA = 0.3283

freqC = 0.0534

freqG = 0.1072

freqT = 0.5110

R(a) [AC] = 1.0000

R(b) [AG] = 8.8964

R(c) [AT] = 0.2994

R(d) [CG] = 0.2994

R(e) [CT] = 9.2786

R(f) [GT] = 1.0000

gamma shape = 1.3690

--

PAUP* Commands Block:

If you want to load the selected model and associated estimates in PAUP*,

attach the next block of commands after the data in your PAUP file:

[!

Likelihood settings from best-fit model (TIM1+G) selected by AIC

with jModeltest 2.1.10 v20160303 on Thu Jul 19 19:01:06 PDT 2018]

BEGIN PAUP;

Lset base=(0.3283 0.0534 0.1072 ) nst=6 rmat=(1.0000 8.8964 0.2994 0.2994 9.2786) rates=gamma shape=1.3690 ncat=4 pinvar=0;

END;

ITS

Model selected:

Model = GTR+G

partition = 012345

-lnL = 7397.5640

K = 141

freqA = 0.2135

freqC = 0.2455

freqG = 0.2629

freqT = 0.2781

R(a) [AC] = 1.6179

R(b) [AG] = 2.4575

R(c) [AT] = 1.5632

R(d) [CG] = 0.6692

R(e) [CT] = 1.9475

R(f) [GT] = 1.0000

gamma shape = 0.5400

--

PAUP* Commands Block:

If you want to load the selected model and associated estimates in PAUP*,

attach the next block of commands after the data in your PAUP file:

[!

Likelihood settings from best-fit model (GTR+G) selected by AIC

with jModeltest 2.1.10 v20160303 on Thu Jul 19 19:00:52 PDT 2018]

BEGIN PAUP;

Lset base=(0.2135 0.2455 0.2629 ) nst=6 rmat=(1.6179 2.4575 1.5632 0.6692 1.9475) rates=gamma shape=0.5400 ncat=4 pinvar=0;

END;

(1) SeqALL_BEAST_exc_meratus_2.xml = Normal


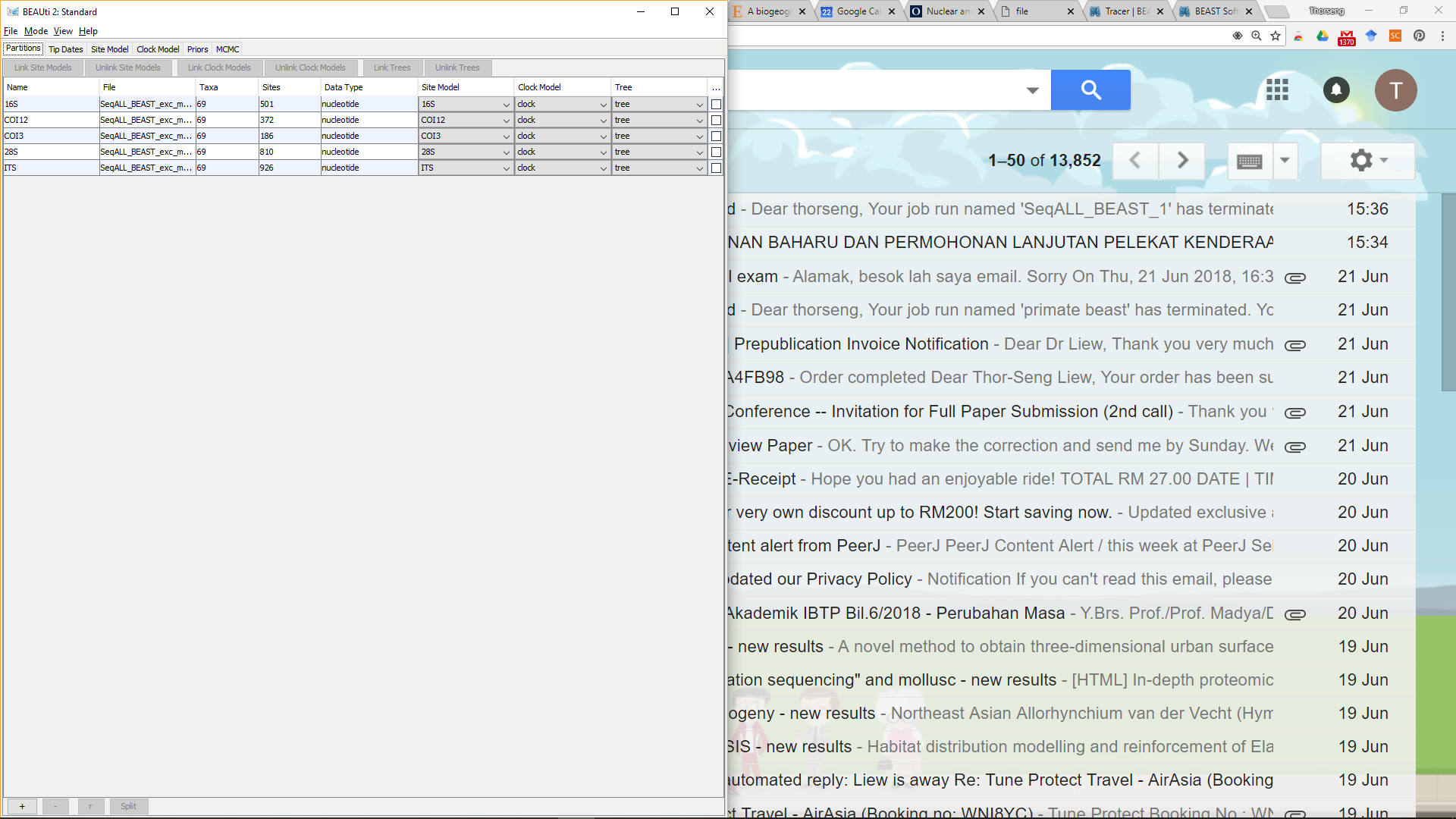


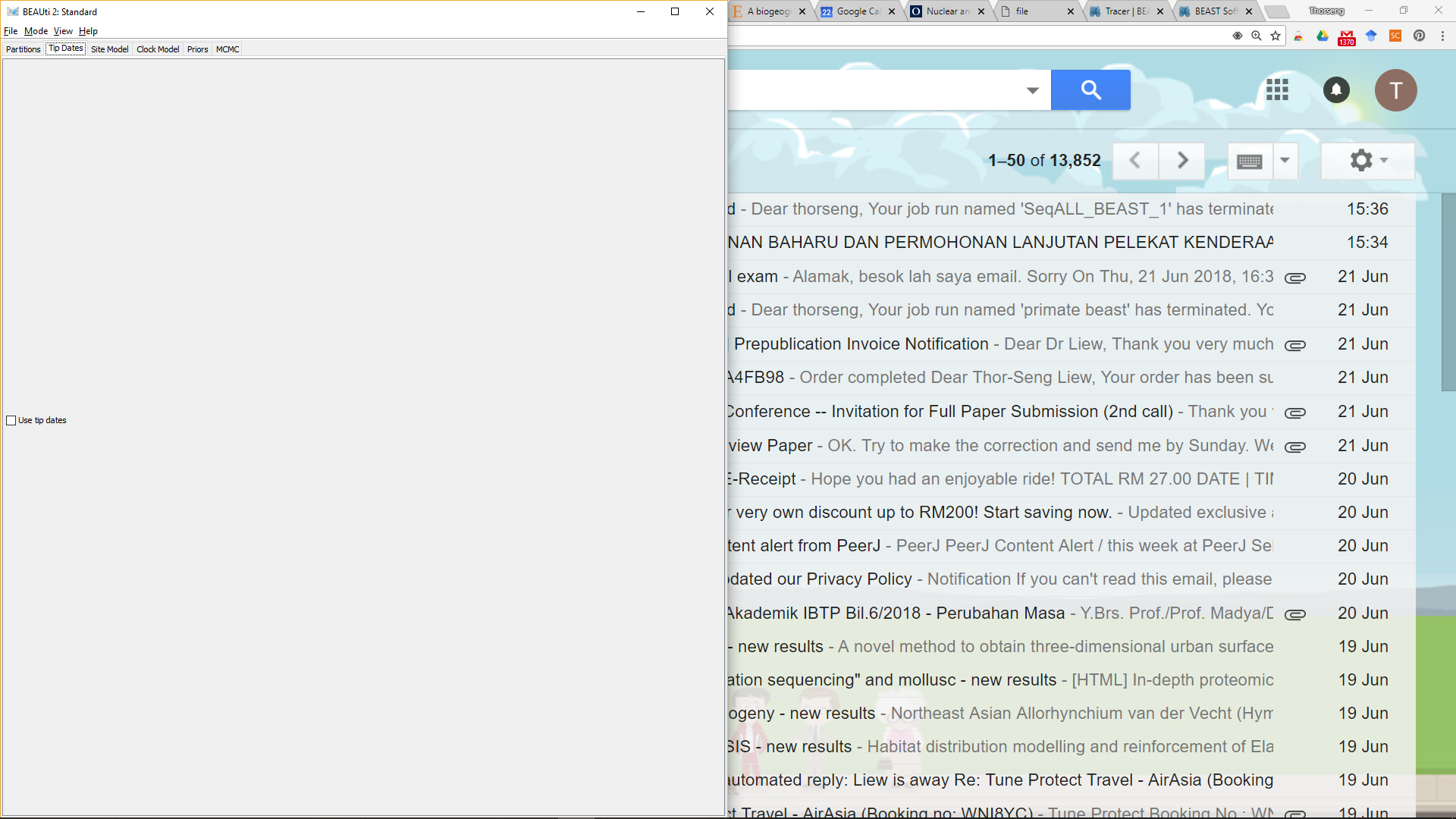


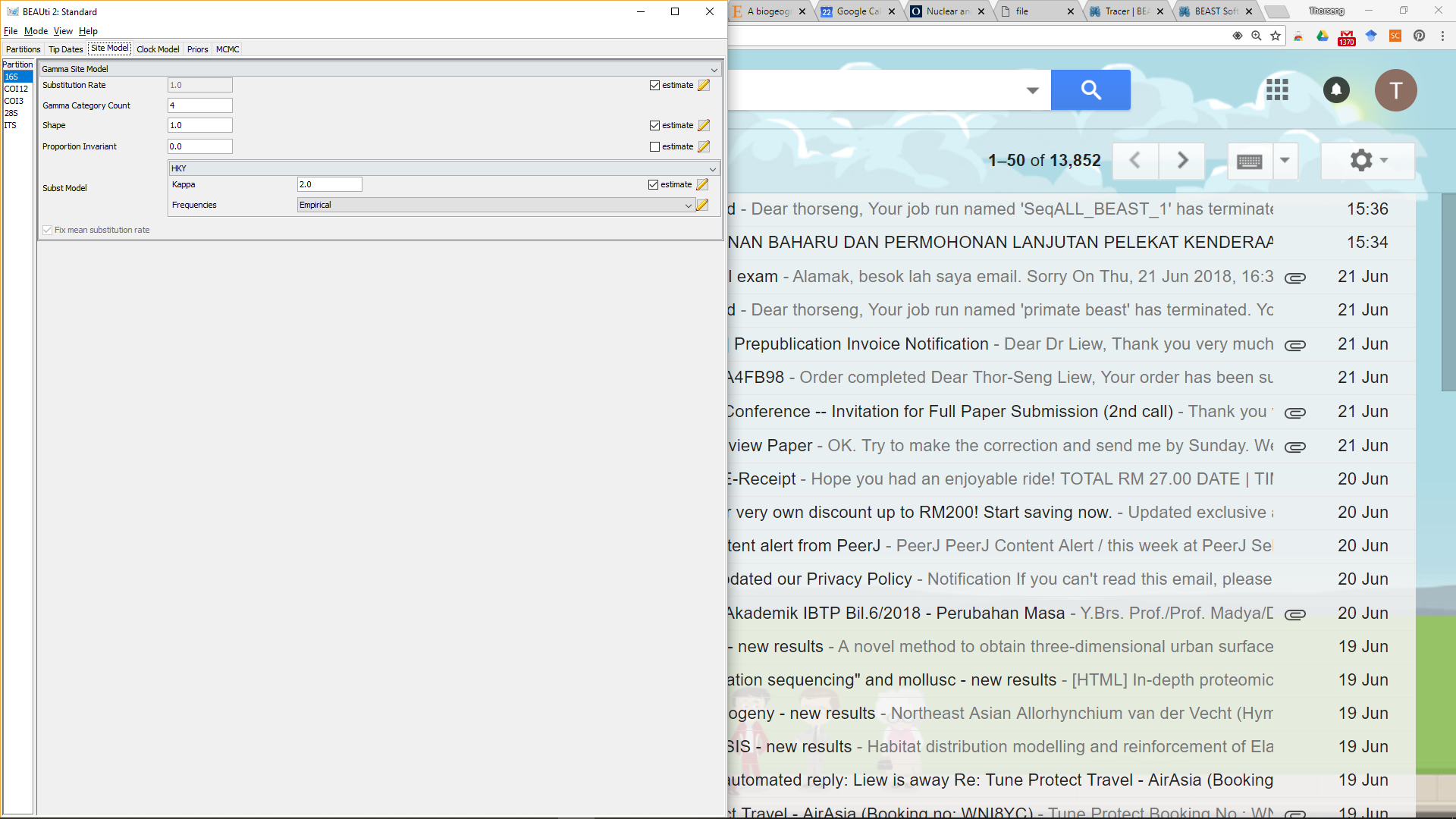


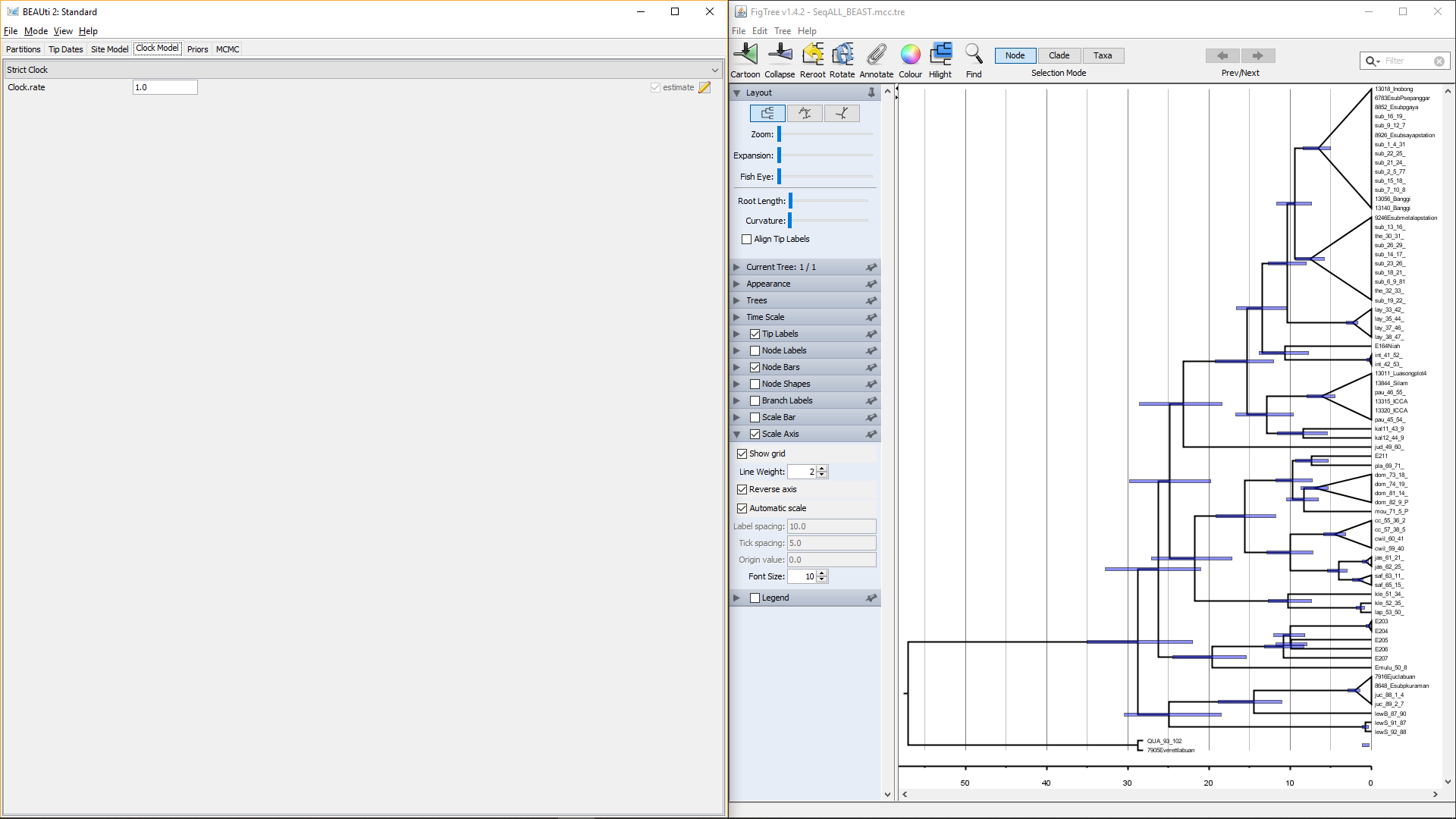


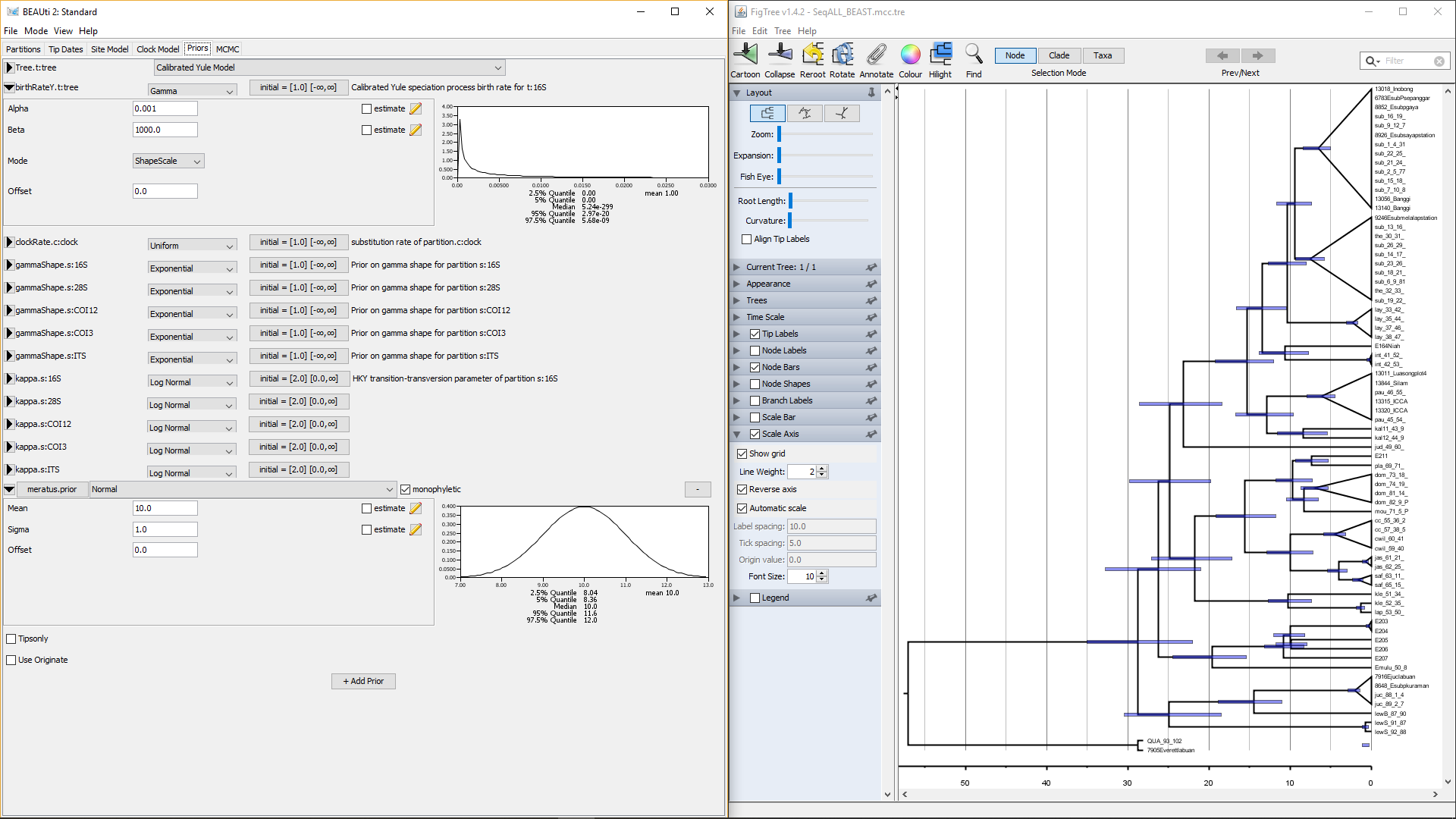


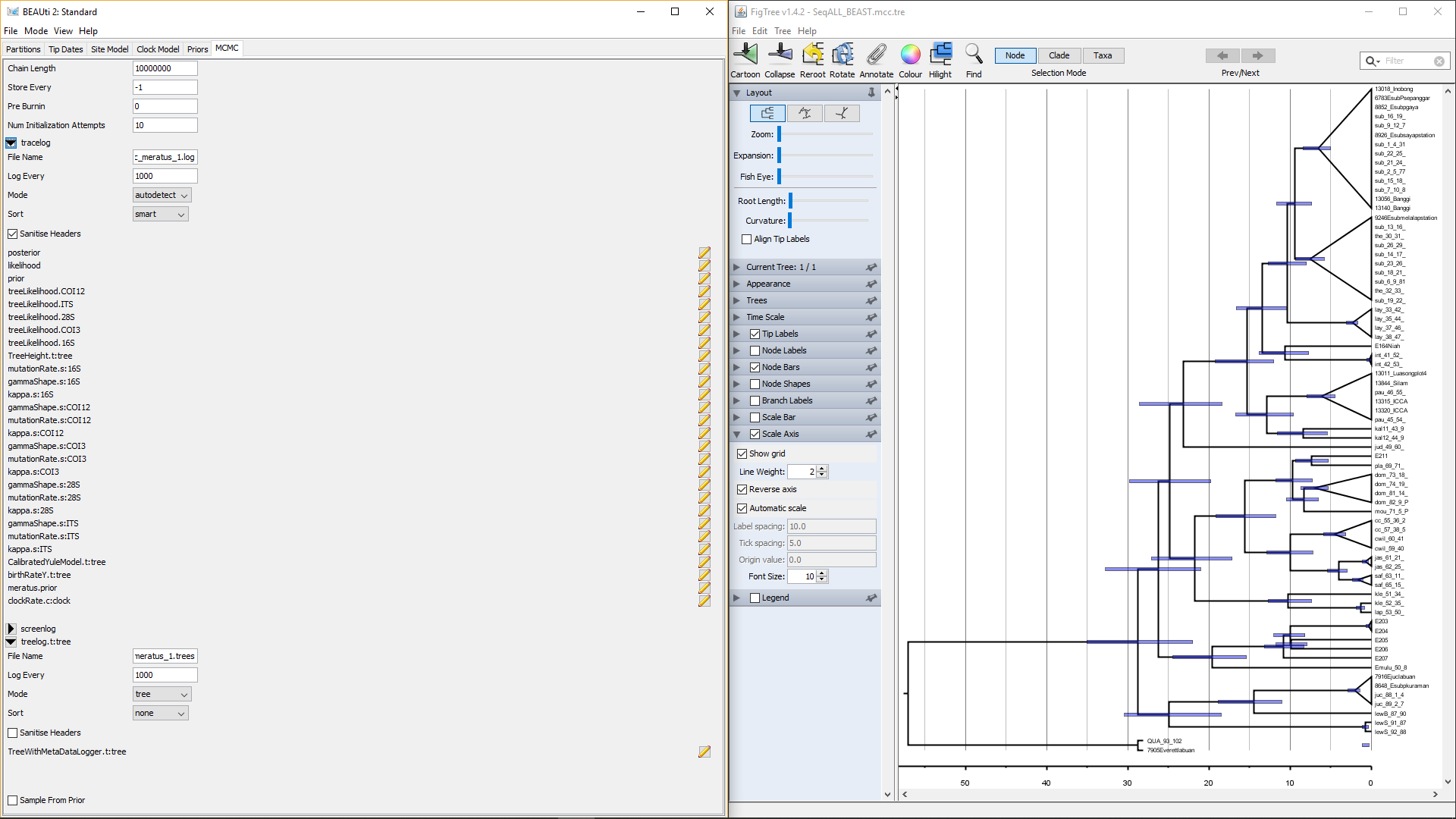


(2) SeqALL_BEAST_exc_meratus_2.xml = clock model unlink – mtDNA & nrDNA


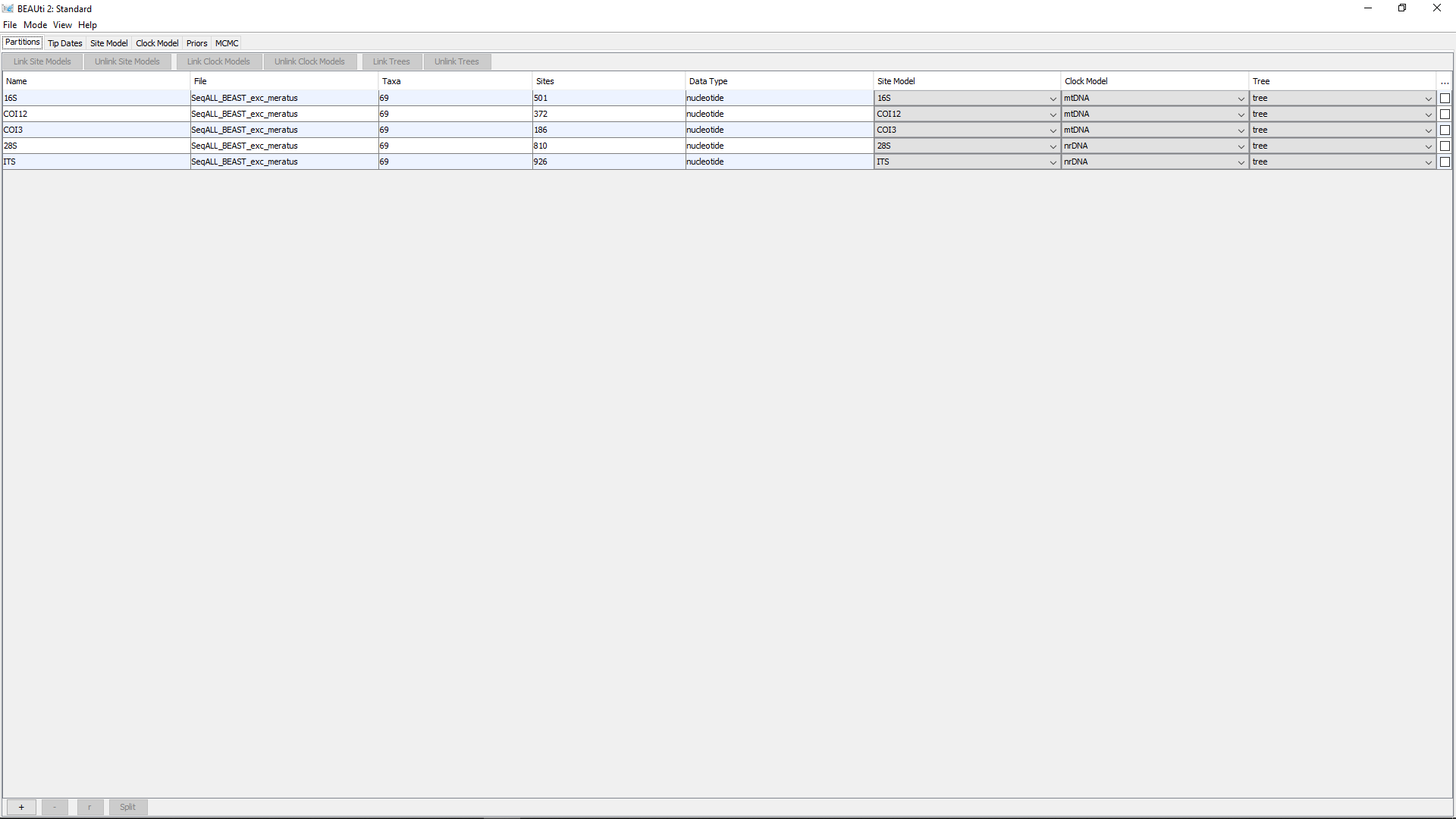


(3) SeqALL_BEAST_exc_meratus_2.xml = clock model unlink – mtDNA & nrDNA & GTR
