# Supplementary material for: Molecular phylogenetics and evolutionary history of the endemic land snail genus Everettia in northern Borneo
Source: PeerJ. 2020 Jul 9;8:e9416. doi: 10.7717/peerj.9416 (PMC7354840; doi:10.7717/peerj.9416)

The BORNEENSIS collection consists of 860 collection lots of *Everettia* species from Sabah that collected between year 2000 and 2018 (Figure X1). Among these collection lots, 758 collection lots of *Everettia* were identified until species level while the remaining of the collection lots are juvenile shells of *Everettia* that cannot be identified into species level. After excluding a collection lot that the exact location cannot be determined, the final distribution data consists of 757 collection lots which consists of 2203 specimens of 17 *Everettia* species from Sabah (Appendix X1).

The sampling bias in the distribution data is negligible. As shown in Figure X1, the collection lots’ distribution for all the *Everettia* species, the entire Sabah have been covered adequately in term of the geographical space with some areas have been sampled denser due to the heterogeneity of the habitat such as mountain ranges and islands.

Appendix X1. Distribution data of 17 *Everettia* species of Sabah in BORNEENSIS collection, Universiti Malaysia Sabah.

Appendix X2. Correlations between bioclim variables.

| Bioclim variables | Other bioclim varialable highly correlated to  (r > 0.8 and P < 0.001) |
| --- | --- |
| BIO1 Annual Mean Temperature | BIO10 Mean Temperature of Warmest Quarter (1.00) BIO11 Mean Temperature of Coldest Quarter (1.00) BIO5 Max Temperature of Warmest Month (0.96) BIO6 Min Temperature of Coldest Month (0.98) BIO8 Mean Temperature of Wettest Quarter (0.99) BIO9 Mean Temperature of Driest Quarter (0.99) |
| BIO7 Temperature Annual Range | BIO2 Mean Diurnal Range (0.92) |
| BIO4 Temperature Seasonality | BIO3 Isothermality (-0.89) |
| BIO12 Annual Precipitation | BIO13 Precipitation of Wettest Month (0.82) BIO14 Precipitation of Driest Month (0.85) BIO16 Precipitation of Wettest Quarter (0.88) BIO17 Precipitation of Driest Quarter (0.87) BIO18 Precipitation of Warmest Quarter (0.81) |
| BIO15 Precipitation Seasonality | None |
| BIO19 Precipitation of Coldest Quarter | BIO13 Precipitation of Wettest Month (0.81) BIO16 Precipitation of Wettest Quarter (0.80) |


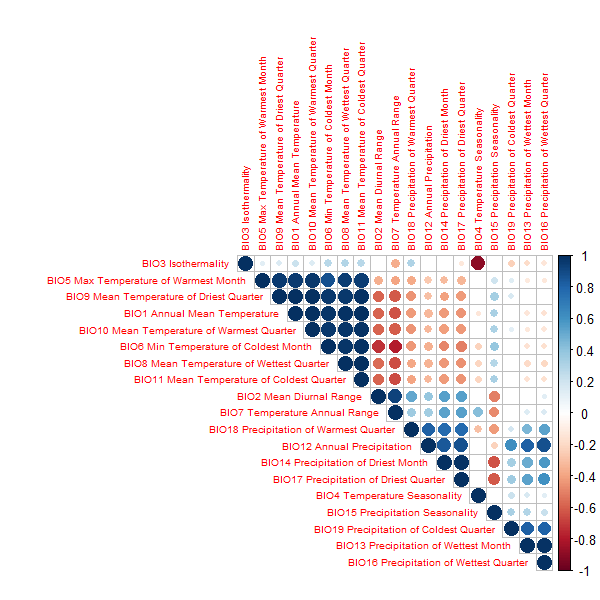


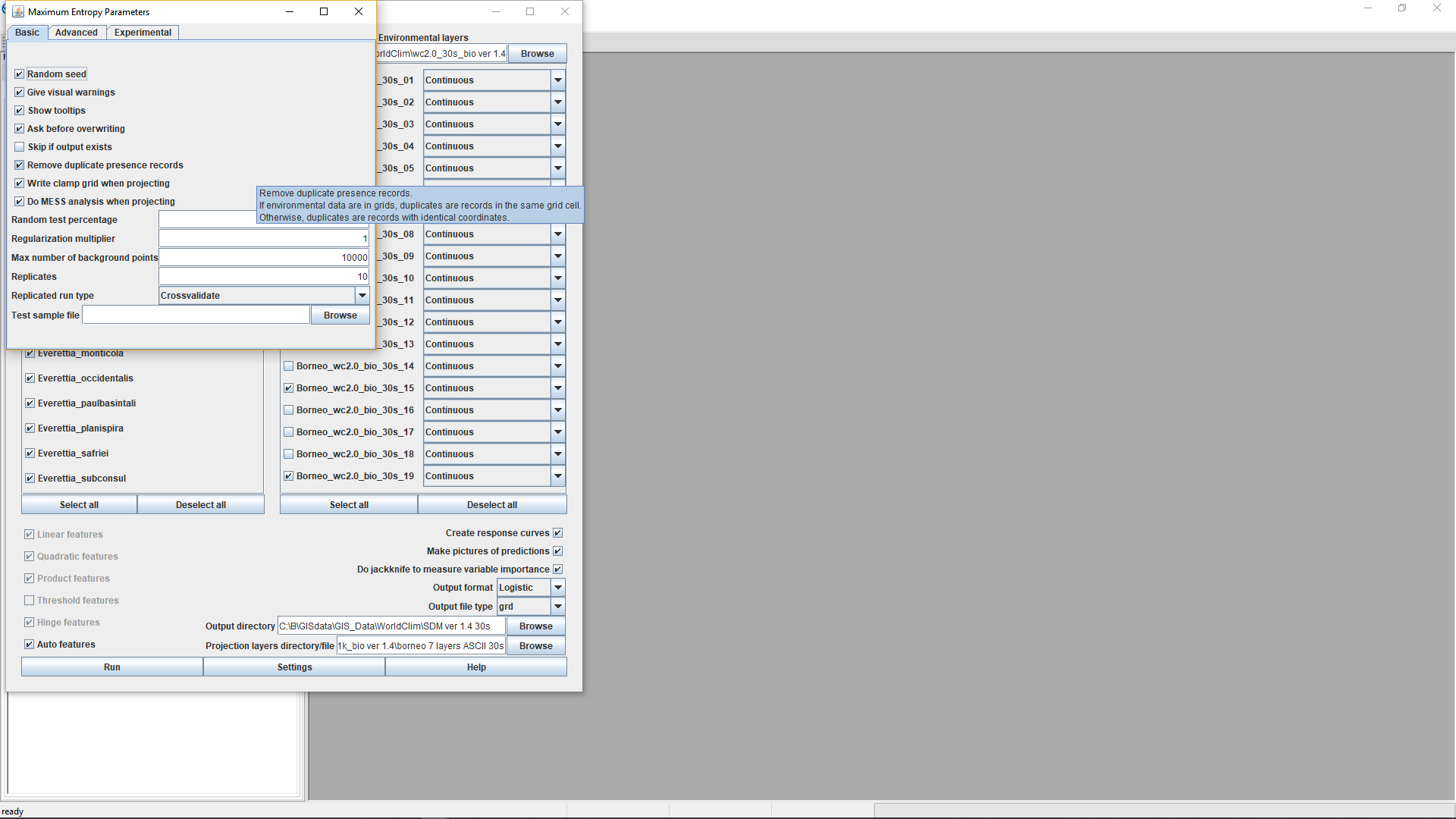


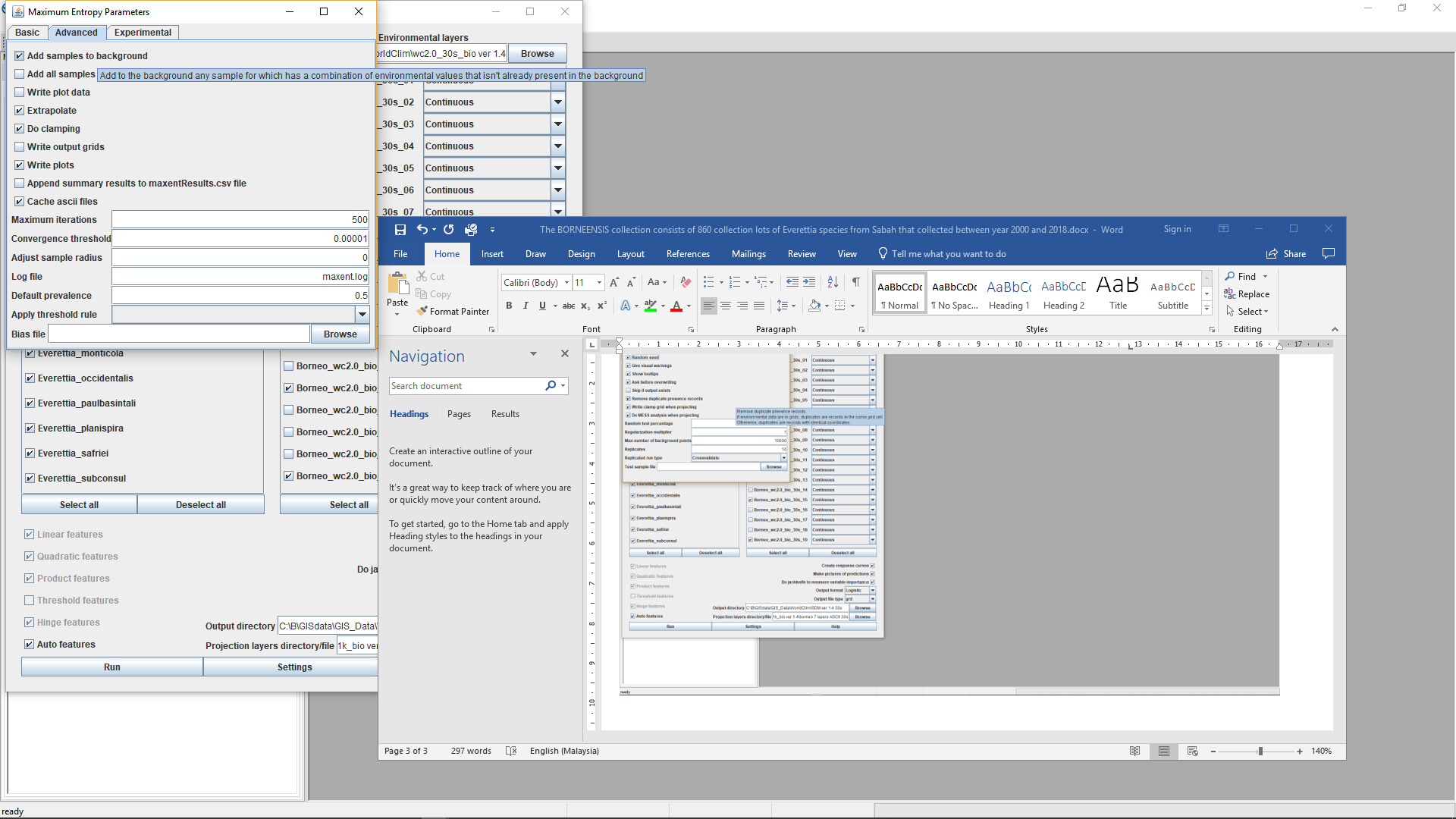


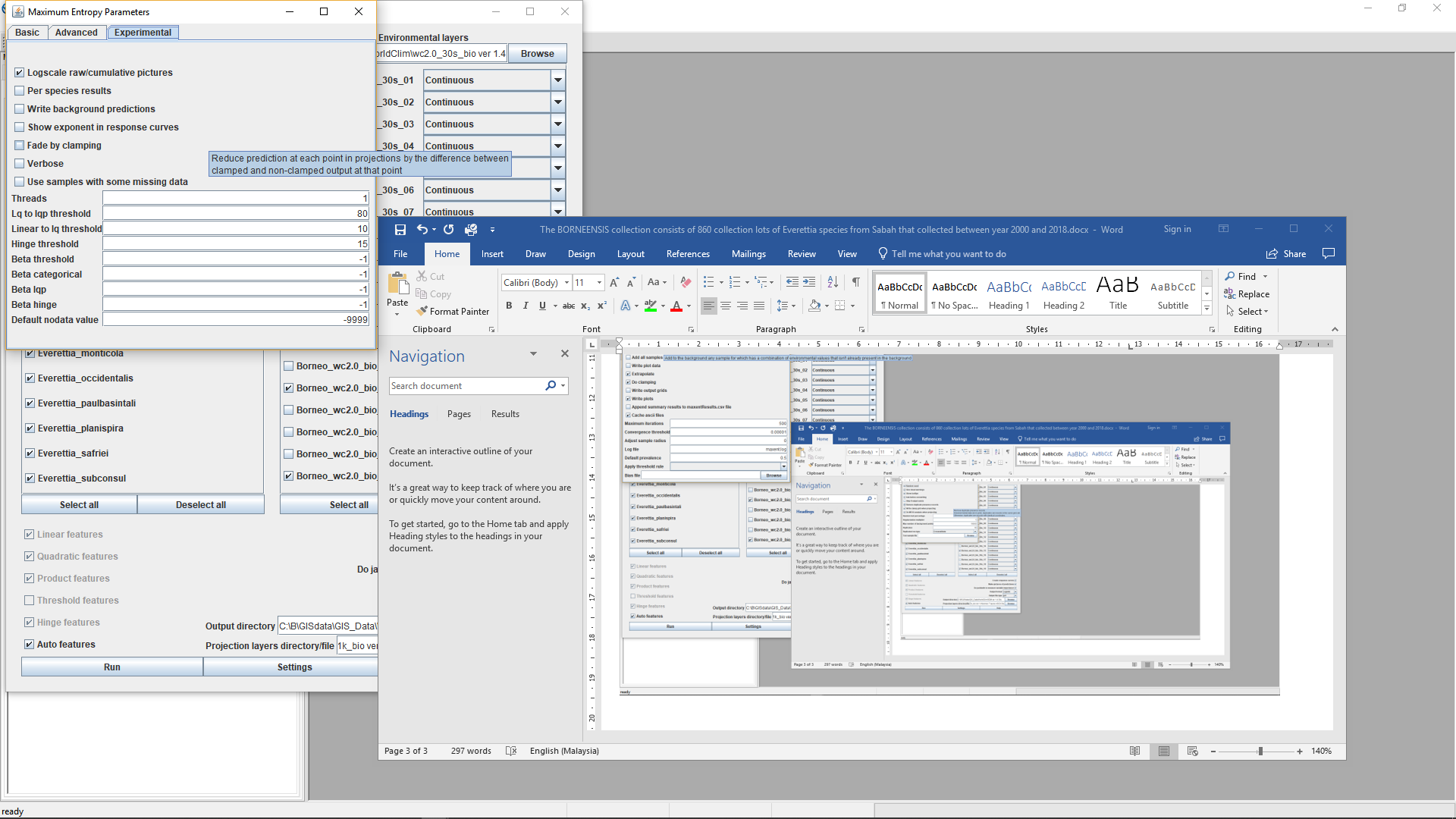


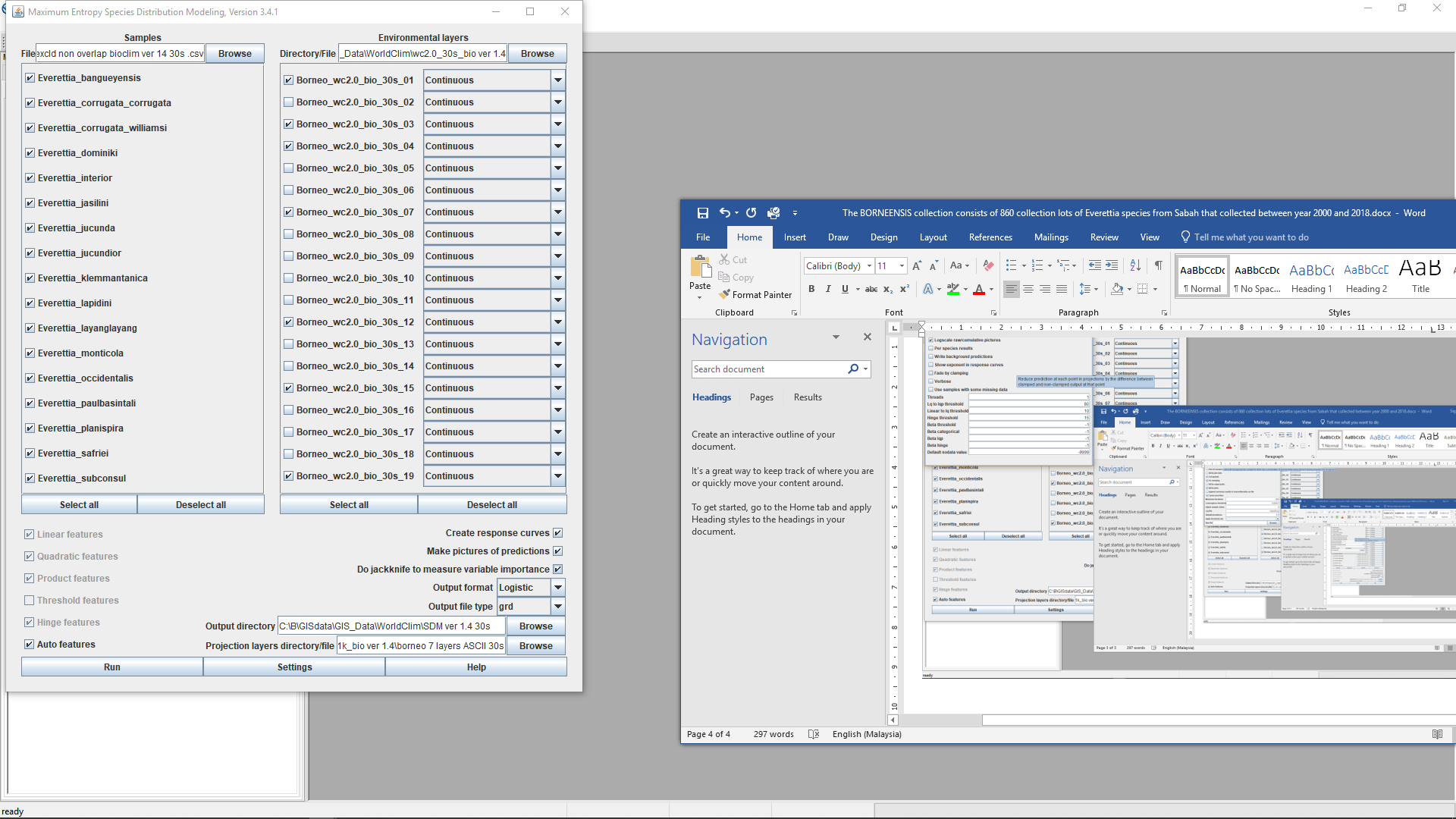

Supplement: Supplemental Information 3 — Bioclimatic variables were sampled 500 at random locations in Borneo in each of the 19 climatic layers. Collinearity among the 19 climatic variables was evaluated by using pairwise Pearson’s r correlation. After the analysis, 12 highly correlated variables (r > 0.8) were excluded from MAXENT analysis. The seven climatic variables were used for species distribution modelling, namely, BIO1 Annual Mean Temperature, BIO3 Isothermality, BIO4 Temperature Seasonality, BIO7 Temperature Annual Range, BIO12 Annual Precipitation, BIO15 Precipitation Seasonality, and BIO19 Precipitation of Coldest Quarter. [file peerj-08-9416-s003.docx]
