# Supplementary material for: Molecular phylogenetics and evolutionary history of the endemic land snail genus Everettia in northern Borneo
Source: PeerJ. 2020 Jul 9;8:e9416. doi: 10.7717/peerj.9416 (PMC7354840; doi:10.7717/peerj.9416)
Supplement: Supplemental Information 5 — The input files and outputs of Bayesian (BA) and Maximum Likelihood (ML) analysis for each of two different best-fit substitution models selected by BIC and AIC criteria, respectively. The phylogenies for each of the analyses were summarised in the word document file. [file peerj-08-9416-s005.zip › Additional File 5/Additional File 5.docx]

**Additional File 5.** A zipped folder consists of input files and outputs of Bayesian (BA) and Maximum Likelihood (ML) analysis for the each of two different best-fit substitution models that selected by BIC and AIC criteria, respectively. The phylogenies for each of the analyses were summarised in the word document file.

**List of the files in the folder:**

1. Bayesian analysis.
   1. Input file for Bayesian analysis – sequence alignment with MrBayes block.
      1. SeqALL with Bayes Block AIC 5 partition.nex
      2. SeqALL with Bayes Block BIC 3 partition.nex
   2. Output file for Bayesian analysis
      1. infile.nex.con.AIC.tre
      2. infile.nex.con.BIC.tre
2. Maximum likelihood analysis
   1. Input file for Maximum likelihood – sequence alignment
      1. See Additional File 4. Concatenated DNA Data Matrix for 16S, COI, ITS, and 28S Sequences for 73 taxa.fas
   2. Input file for Maximum likelihood – partition files
      1. iq_tree.partition AIC.nex
      2. iq_tree.partition BIC.nex
   3. Output file for Maximum likelihood analysis
      1. ML_analysis_3_partition_BIC.tre
      2. ML_analysis_5_partition_AIC.tre


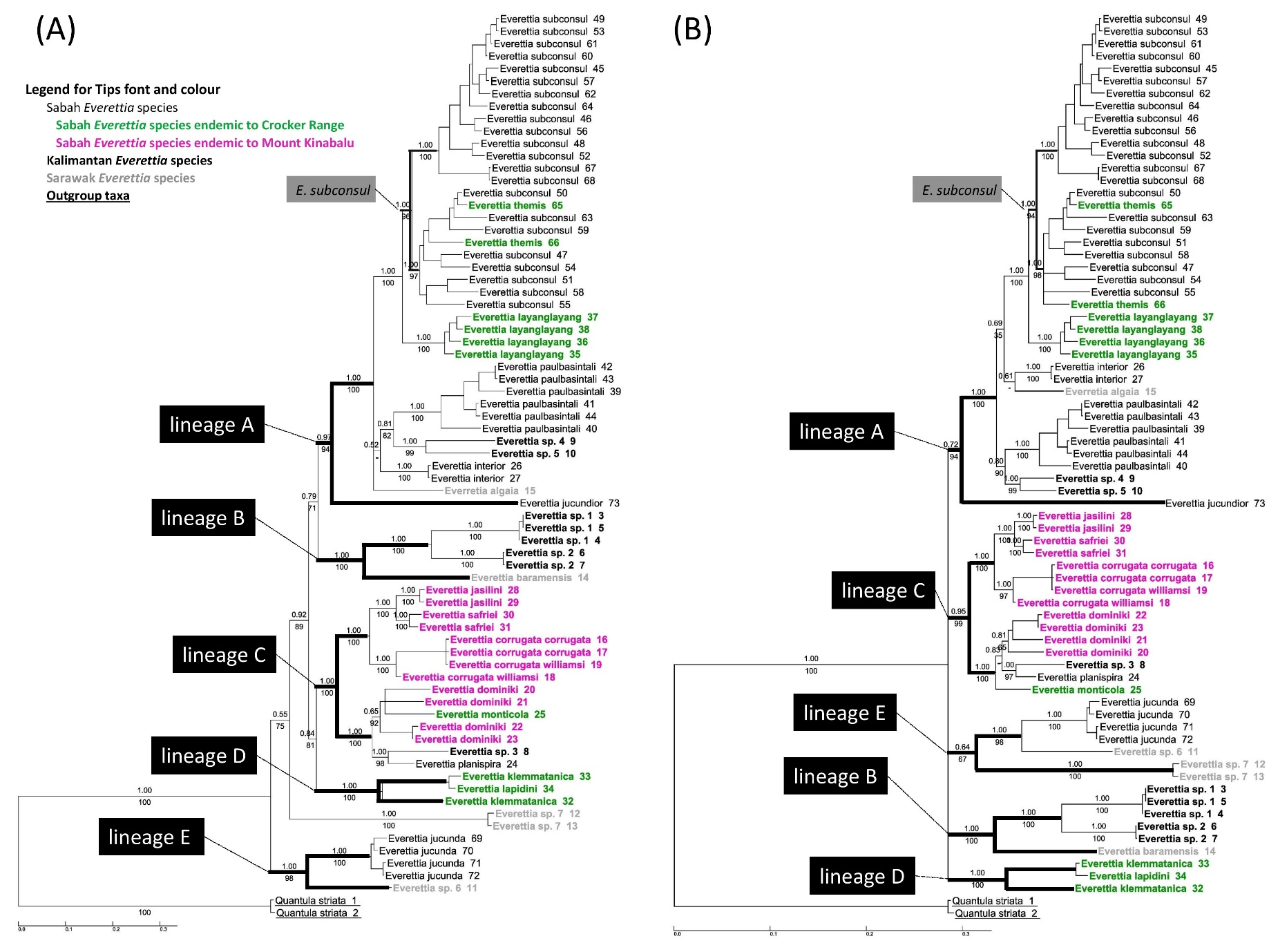


Figure S1. The phylogeny of 25 *Everettia* species with *Quantula striata* as outgroup. Bayesian inference 50% majority-rule consensus trees based on the concatenated dataset consisting of parts of 28S, ITS-1, COI, and 16S. Bayesian (BA) and Maximum Likelihood (ML) analysis for the each of two different best-fit substitution models selected by BIC (A) and AIC (B) criteria. Bayesian posterior probabilities and bootstrap support after 1000 maximum likelihood replicates are shown above and below the branches of the nodes. The font and colour of the taxa name on the tree indicate the distribution of the species. The number after the taxa name specimen number of Table 1, Figs 1 and 2.
