# Supplementary material for: Molecular phylogenetics and evolutionary history of the endemic land snail genus Everettia in northern Borneo
Source: PeerJ. 2020 Jul 9;8:e9416. doi: 10.7717/peerj.9416 (PMC7354840; doi:10.7717/peerj.9416)
Supplement: Supplemental Information 6 — A total of four BEAST input XML files for the combinations two different best-fit substitution models (selected by BIC and AIC criteria) and two calibrated models (Yule model vs. Birth-Death model). The calibrated phylogenies for each of the four analyses were summarised in the word document file. [file peerj-08-9416-s006.zip › Additional File 6/Additional File 6.docx]

**Additional File 6.** A zipped folder consists of input files and outputs of BEAST analysis. There are four BEAST input XML files, and each of the input files is the combination either one of the two different best-fit substitution models (selected by BIC vs AIC criteria) and either one fo the two calibrated models (Yule model vs Birth-Death model). Figure S1 to Figure S4 represent the calibrated phylogenies for each of the four analyses. Table S1 summarise the divergence time of the major lineages of the four different analyses.

**List of the files in the folder:**

1. BEAST analysis.
   1. Input file for BEAST analysis – xml file.
      1. BEAST_BD_AIC_39taxa_allgenes_.xml
      2. BEAST_BD_BIC_39taxa_allgenes.xml
      3. BEAST_YULE_AIC_39taxa_allgenes_.xml
      4. BEAST_YULE_BIC_39taxa_allgenes.xml
   2. Output file for Bayesian analysis
      1. infile.nex.con.AIC.tre
      2. infile.nex.con.BIC.tre

**Table S1.** Divergent time estimates (mean and 95% Highest Posterior Density (HPD) interval in a million years ago, Mya) for the four combinations of either one of the two different best-fit substitution models (selected by BIC vs AIC criteria) and either one fo the two calibrated models (Yule model vs Birth-Death model).

| Major phylogenetic lineages of *Everettia* species in Sabah | BIC + Birth-Death model  (Figure S1) | AIC + Yule model  (Figure S2) | BIC + Birth-Death model  (Figure S3) | AIC + Yule model  (Figure S4) |
| --- | --- | --- | --- | --- |
| lineage A | 20.0 (13.5-27.3) | 21.9 (15.1 – 29.9) | 19.9 (13.5 – 27.1) | 20.2 (11.6 – 30.3) |
| lineage B | 17.0 (12.5 – 22.1) | 17.3 (12.6 – 22.8) | 16.9 (12.5 – 22.1) | 17.3 (11.6 – 24.5) |
| lineage C | 15.5 (9.9 – 21.6) | 15.3 (9.7 – 21.7) | 15.3 (9.9 – 21.5) | 15.5 (8.2 – 23.9) |
| lineage D | 9.6 (5.2 – 14.4) | 9.6 (5.1- 14.5) | 9.6 (5.3 – 14.5) | 9.5 (3.7 – 16.1) |
| lineage E | 14.0 (7.2 – 21.7) | 13.4 (7.2 – 20.7) | 13.9 (7.4 – 21.5) | 13.0 (5.5 – 22.4) |

**Table S2.** Divergent time estimates (mean in a million years ago, Mya) for the four independent run of best-fit substitution models selected by BIC and calibrated models Birth-Death model.

| Major phylogenetic lineages of *Everettia* species in Sabah | BIC + Birth-Death model  Run No. 1 | BIC + Birth-Death model  Run No. 2 | BIC + Birth-Death model  Run No. 3 | BIC + Birth-Death model  Run No. 4 |
| --- | --- | --- | --- | --- |
| lineage A | 19.96 | 20.03 | 19.89 | 19.99 |
| lineage B | 16.96 | 16.99 | 16.95 | 16.96 |
| lineage C | 15.35 | 15.47 | 15.34 | 15.38 |
| lineage D | 9.58 | 9.62 | 9.58 | 9.59 |
| lineage E | 13.87 | 13.98 | 14.03 | 13.82 |


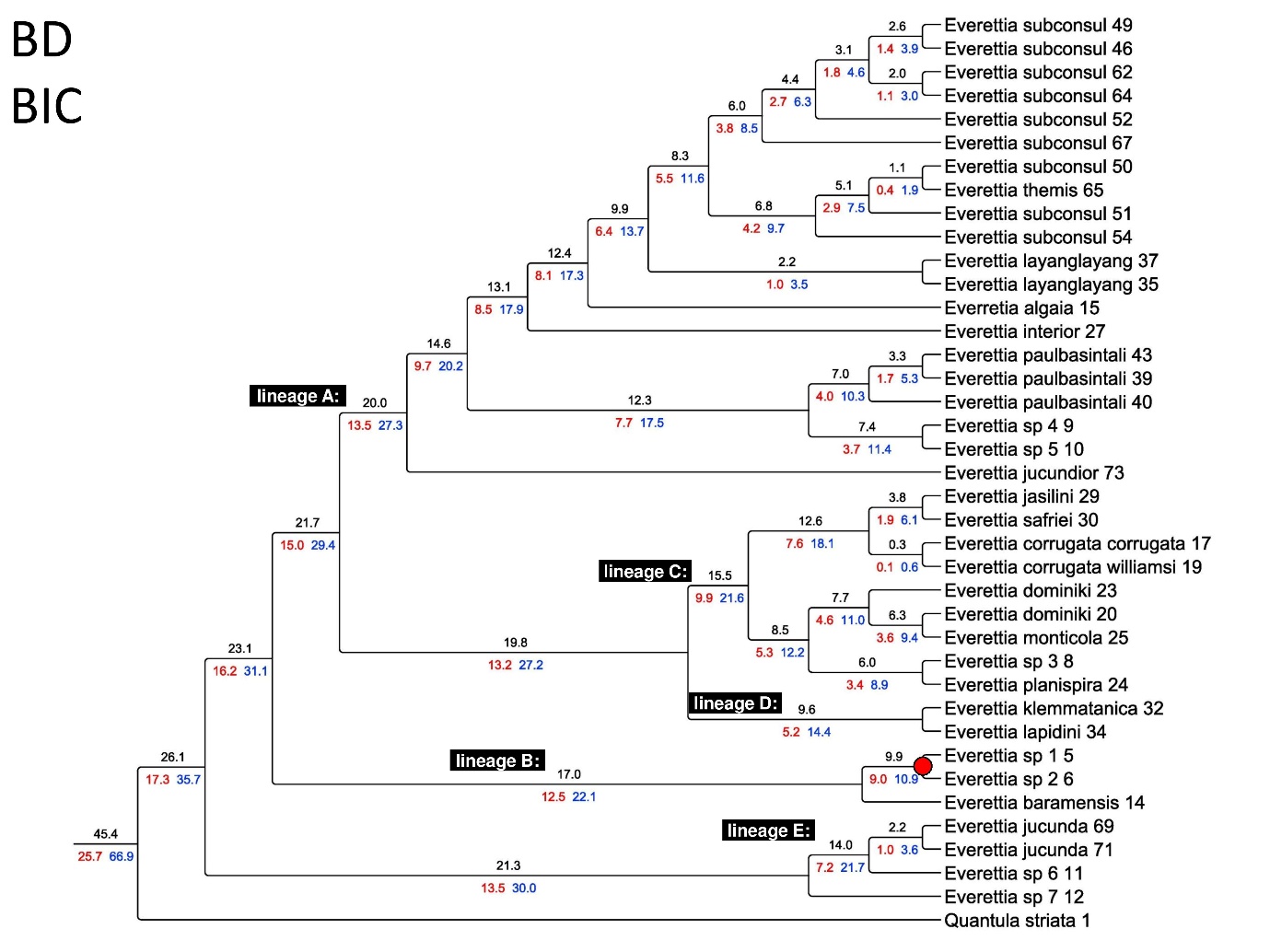


Figure S1. The chronogram for *Everettia*species in Borneo obtained from divergence time estimation using BEAST based on the best-fit model selected under BIC and calibrated Birth-Death model. The divergence times (in a million years ago, Mya) of the major lineages are shown as values on the chronogram branches: above branch values are mean ages and below branch values the 95% Highest Posterior Density (HPD) interval (minimum in red colour text, and maximum in the blue colour text). The red point indicates the calibration points. The number after the taxa name specimen number of Table 1, Figs 1 and 2.


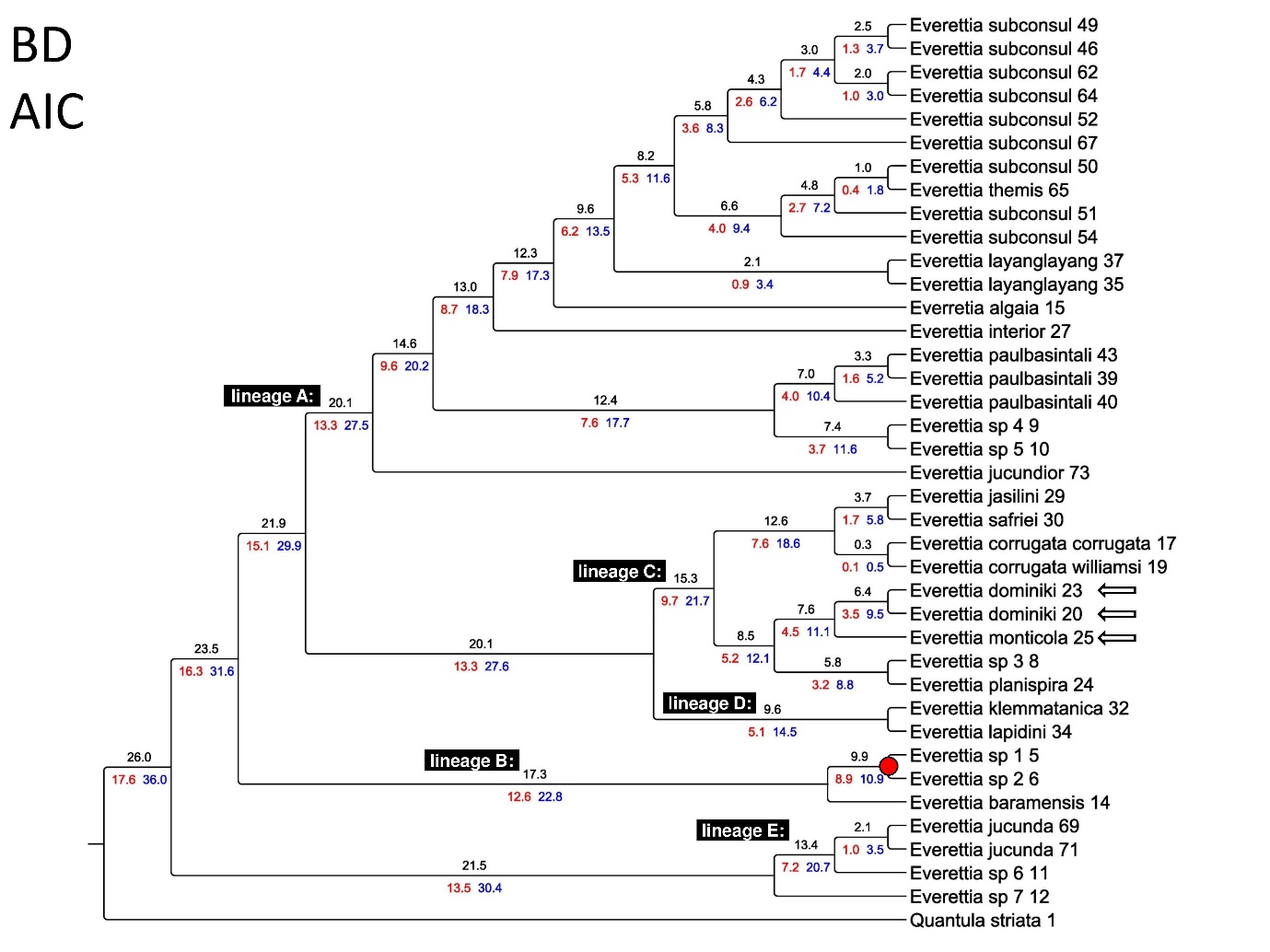


Figure S2. The chronogram for *Everettia*species in Borneo obtained from divergence time estimation using BEAST based on the best-fit model selected under AIC and calibrated Birth-Death model. The divergence times (in a million years ago, Mya) of the major lineages are shown as values on the chronogram branches: above branch values are mean ages and below branch values the 95% Highest Posterior Density (HPD) interval (minimum in red colour text, and maximum in the blue colour text). The red point indicates the calibration points. The number after the taxa name specimen number of Table 1, Figs 1 and 2. Arrows next to taxa name indicate the parts of phylogeny that differs from Figure S1.


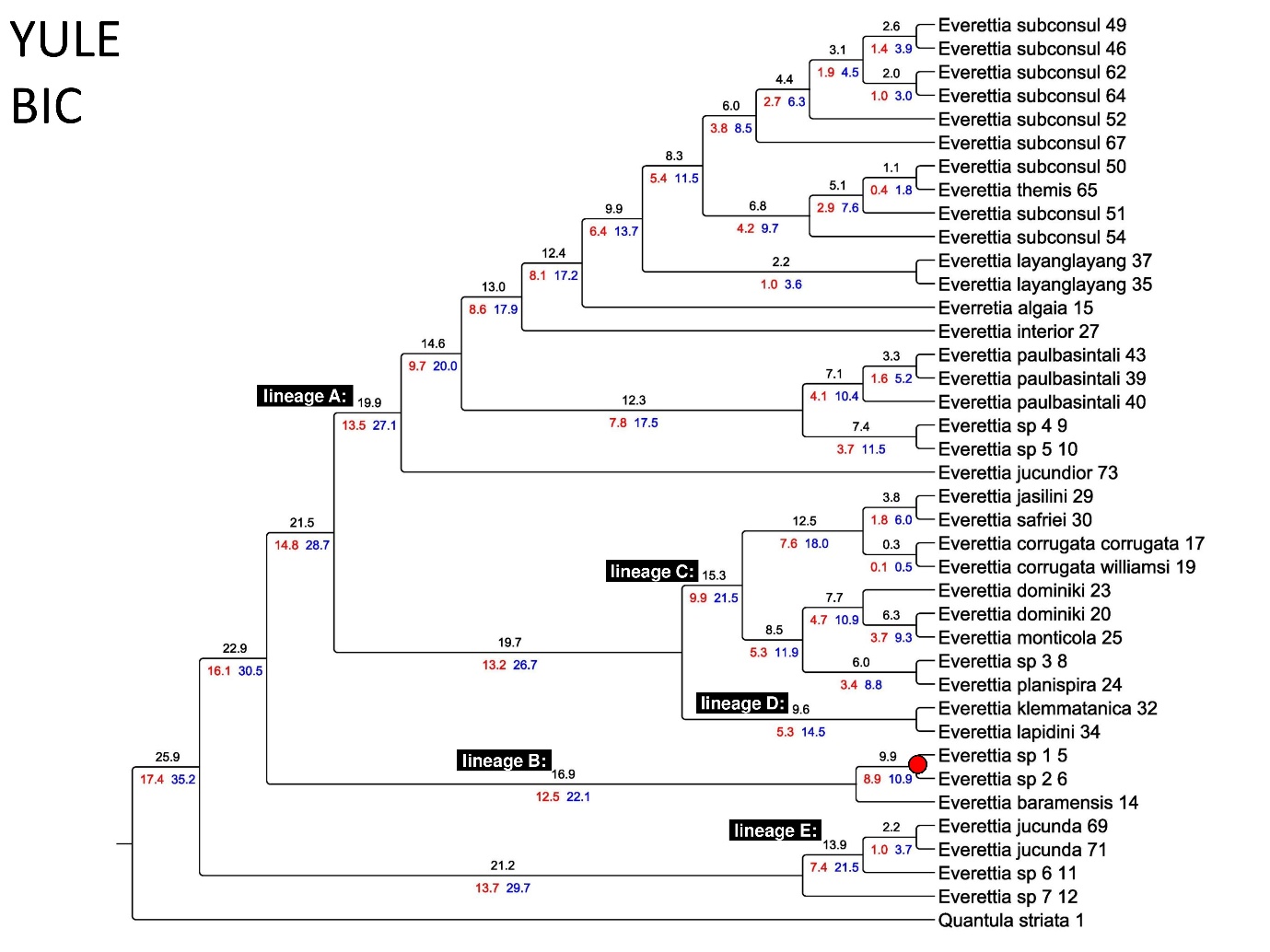


Figure S3. The chronogram for *Everettia*species in Borneo obtained from divergence time estimation using BEAST based on the best-fit model selected under BIC and calibrated Yule model. The divergence times (in a million years ago, Mya) of the major lineages are shown as values on the chronogram branches: above branch values are mean ages and below branch values the 95% Highest Posterior Density (HPD) interval (minimum in red colour text, and maximum in the blue colour text). The red point indicates the calibration points. The number after the taxa name specimen number of Table 1, Figs 1 and 2. Arrows next to taxa name indicate the parts of phylogeny that differs from Figure S1.


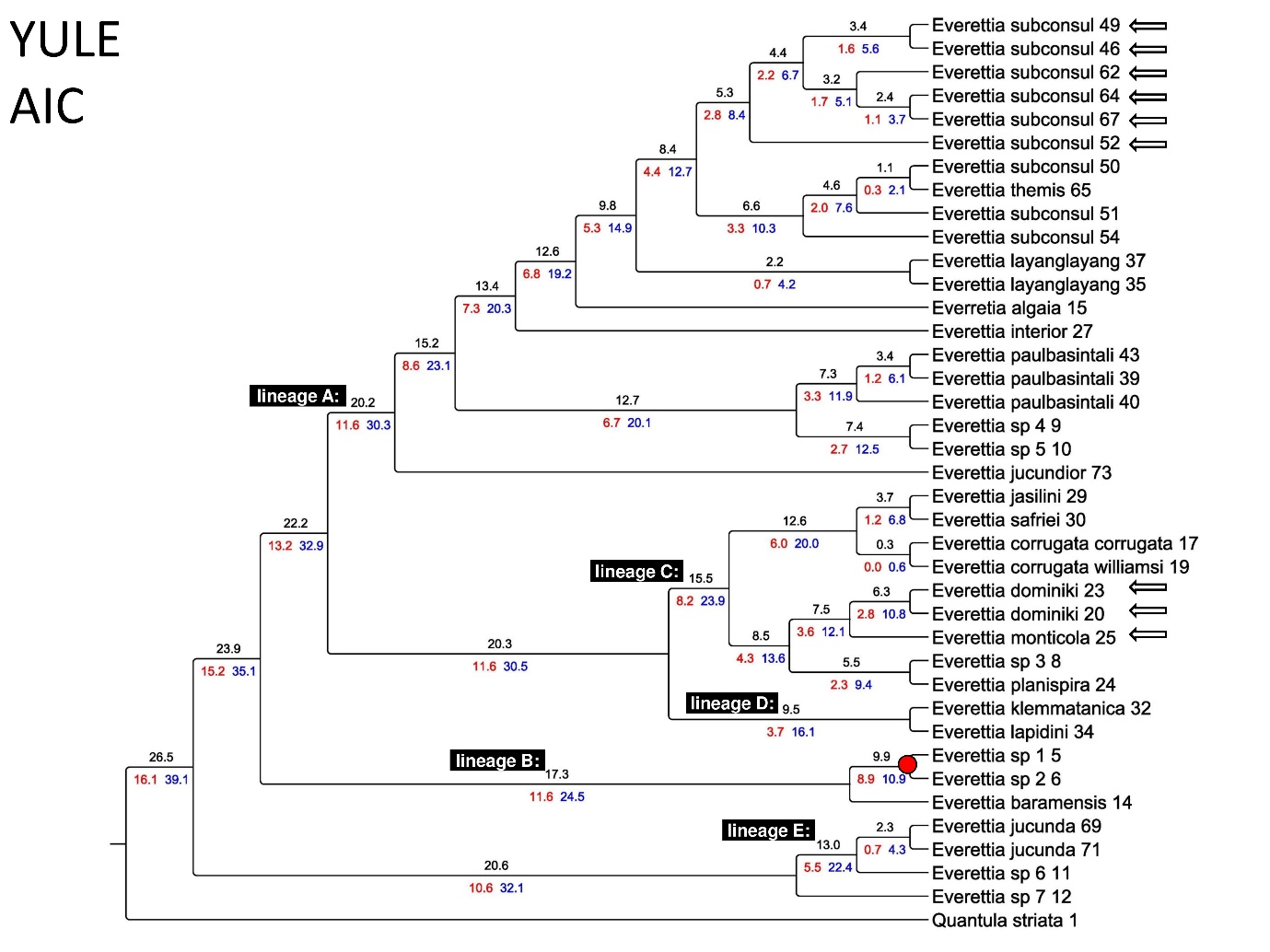


Figure S4. The chronogram for *Everettia*species in Borneo obtained from divergence time estimation using BEAST based on the best-fit model selected under AIC and calibrated Yule model. The divergence times (in a million years ago, Mya) of the major lineages are shown as values on the chronogram branches: above branch values are mean ages and below branch values the 95% Highest Posterior Density (HPD) interval (minimum in red colour text, and maximum in the blue colour text). The red point indicates the calibration points. The number after the taxa name specimen number of Table 1, Figs 1 and 2. Arrows next to taxa name indicate the parts of phylogeny that differs from Figure S1.
